# Anti-Donor Immune Responses Elicited by Allogeneic Mesenchymal Stem Cells and Their Extracellular Vesicles: Are We Still Learning?

**DOI:** 10.3389/fimmu.2017.01626

**Published:** 2017-11-24

**Authors:** Paul Lohan, Oliver Treacy, Matthew D. Griffin, Thomas Ritter, Aideen E. Ryan

**Affiliations:** ^1^Regenerative Medicine Institute (REMEDI), School of Medicine, College of Medicine, Nursing and Health Sciences, National University of Ireland Galway, Galway, Ireland; ^2^Discipline of Pharmacology and Therapeutics, School of Medicine, College of Medicine, Nursing and Health Sciences, National University of Ireland Galway, Galway, Ireland; ^3^CURAM Centre for Research in Medical Devices, National University of Ireland, Galway, Ireland

**Keywords:** allogeneic, allo-mesenchymal stromal cell, anti-donor immune response, immunogeniciy, inflammation, immunomodulation

## Abstract

Mesenchymal stromal cells (MSC) have been used to treat a broad range of disease indications such as acute and chronic inflammatory disorders, autoimmune diseases, and transplant rejection due to their potent immunosuppressive/anti-inflammatory properties. The breadth of their usage is due in no small part to the vast quantity of published studies showing their ability to modulate multiple immune cell types of both the innate and adaptive immune response. While patient-derived (autologous) MSC may be the safer choice in terms of avoiding unwanted immune responses, factors including donor comorbidities may preclude these cells from use. In these situations, allogeneic MSC derived from genetically unrelated individuals must be used. While allogeneic MSC were initially believed to be immune-privileged, substantial evidence now exists to prove otherwise with multiple studies documenting specific cellular and humoral immune responses against donor antigens following administration of these cells. In this article, we will review recent published studies using non-manipulated, inflammatory molecule-activated (licensed) and differentiated allogeneic MSC, as well as MSC extracellular vesicles focusing on the immune responses to these cells and whether or not such responses have an impact on allogeneic MSC-mediated safety and efficacy.

## Introduction

Mesenchymal stem/stromal cells (MSC) are a readily accessible cell source in which interest has expanded hugely in the last two decades (1). The allogeneic use of these cells was touted as being safe owing to their low expression of major histocompatibility complex (MHC) and co-stimulatory molecules and the fact that they can suppress the activity of numerous immune cell populations (2–4). However, in more recent years several groups have shown in different models that MSC do induce anti-donor immune responses from different facets of the immune system, including dendritic cells, T cells, and B-cell mediated allo-antibodies (4–7). Despite the fact that allogeneic MSC can be recognized by the host immune system, administration of these cells to human subjects has been shown, for the most part, to be safe in several different disease settings (5, 8–10). There are a small number of studies describing several treatment emergent adverse events (TEAE) following administration of decidual stromal cells or adipose-derived stromal cells to patients, however, it appears that these are unrelated to the development of anti-donor immune responses (10–13). However, the field should remain vigilant to adverse immune responses which may have detrimental effects in the long term or if the patient receives a transplant later in life which may be rejected as a result of a pre-existing anti-donor memory response, either cellular or humoral (5). To this end, here we have critically assessed recent allogeneic MSC literature as a follow up to our previous review with a focus on studies which have investigated the immune response to allogeneic MSC either *in vitro, in vivo*, or as part of a clinical trial to update the field on the potential responses to these cells (5).

## Immunogenicity and Immunomodulatory Potential of Allogeneic Non-Manipulated MSC

The advantages of using allogeneic over autologous MSC have been well documented with perhaps the most compelling advantage being the ability to obtain cells from healthy donors followed by expansion *in vitro* to clinically relevant numbers. The cells can then be banked and transported where required with minimal delay. Another commonly touted advantage of allogeneic MSC is their low immunogenicity and while there may be some truth in this when compared to other allogeneic cell types, emerging evidence suggests that allogeneic MSC can indeed induce a strong immune response *in vivo* which may have severe consequences depending on the disease indication for which the cells are being administered (14). Even *in vitro*, it has been shown that stimulating MHC class II negative equine MSC with interferon (IFN)-γ for 4 days leads to markedly increased expression of MHC class II (15, 16). One could reasonably assume that, in an allogeneic *in vivo* setting, administered MSC would encounter significant levels of IFN-γ which may impair their efficacy, depending on the indication. A study by Joswig et al. (17) assessed the clinical response to repeated intra-articular injections of bone marrow-derived equine allogeneic versus autologous MSC. Clinical parameters assessed in the study showed no differences after the first injection of either autologous or allogeneic MSC. However, following the second injection, a significant adverse response of the joint was seen in horses treated with allogeneic compared to autologous MSC, evidenced by elevated synovial total nucleated cell counts. In the same model, another recent study could demonstrate that anti-sera collected from horses injected with MHC-mismatched MSC contained antibodies that caused the death of equine leukocyte antigen-A2 haplotype MSC in cytotoxicity assays (4, 18). By contrast, Ardanaz and colleagues (19) reported no persistent unwanted effects, including the absence of a hypersensitivity response, following single or repeated intra-articular injections of equine allogeneic MSC from pooled donors. In this study, only a transitory inflammatory response was observed which resolved after 10 days.

Owing to the similarities between the immune systems of humans and non-human primates, immunological analysis of allogeneic MSC in these pre-clinical animal models may have important implications from a clinical perspective. Recently, Isakova and co-workers (20) performed such a study. The aim of the study was to evaluate the immune response caused by intra-cranial injection of allogeneic compared to autologous MSC in rhesus macaques. The authors detected clear signs of allo-recognition as evidenced by significantly higher levels of circulating allo-specific antibodies in serum of macaques that received allogeneic compared to autologous MSC. They also demonstrated, in *in vitro* co-cultures, that peripheral blood mononuclear cells (PBMCs) isolated from allogeneic but not autologous MSC recipients were capable of lysing their respective donor MSC. Furthermore, higher levels of peripheral blood-derived natural killer, B and T cell subsets were recorded in allogeneic MSC recipients with the overall magnitude of the allo-immune response determined by the level of mismatch between the MSC donor and recipient. A detailed understanding of the consequences of these reported changes in immune profiles following allo-MSC therapy is likely to advance the field even further.

Another emerging concept in relation to allo-MSC therapy that should be considered is the idea that allo-MSC cell death following administration may play a role in regulation of ensuing immune responses. Immune responses to dying cells can be affected by multiple factors related to the type of cell death (21). These include, but are not limited to, the cell death pathway, the way dead cells are cleared by innate immune effectors, the nature and phenotype of the effector immune cells, the location of cell clearance, and the immune cell effectors that eventually encounter the antigens presented along with the dead cells (21). Another key consideration is whether to use fresh or cryopreserved cells, as this may impact cell viability and consequent immune responses (22). Indeed, a recent report by Chinnadurai and colleagues demonstrated that cryopreserved MSC were susceptible to T cell-mediated apoptosis (23). In the context of allo-MSC therapy, consideration of all of these issues is likely to shed light on the impact of death of allo-MSC on the immune system, and the implications for the potential of allo-MSC therapeutic efficacy.

## Immunogenicity and Immunomodulatory Potential of Licensed Allogeneic MSC

Few studies have investigated the effects of licensing (treatment with pro-inflammatory stimuli *in vitro*) on allogeneic MSC, particularly *in vivo*. This is most likely because these cells will be targeted by recipient immune cells upon their first encounter in an *in vivo* setting and will receive an inflammatory stimulus, thereby, potentially, negating the need to pre-activate these cells *in vitro*. However, there are some reports using this strategy with the focus primarily being to further enhance the cells’ immunosuppressive ability, as opposed to investigating whether immunogenicity has been increased. In one such study, Mancheno-Corvo and colleagues assessed the ability of adipose-derived MSC (ASC) to inhibit allogeneic T cell proliferation. They correctly pointed out that the majority of similar studies activate T cells at the same time the cells first encounter the MSC in *in vitro* co-culture assays and that such an approach may not be relevant to typical clinical scenarios in which MSC, administered after the onset of symptoms associated with inflammatory disorder, such as Crohn’s disease or rheumatoid arthritis, will encounter and interact with immune cells (e.g., T cells) that are already activated. Upon analysis, they found that ASC co-cultured with T lymphocytes that had been pre-activated for 48 h beforehand had impaired immunosuppressive capacity. However, this ability to inhibit allogeneic T lymphocyte proliferation was restored when ASC were pre-activated with IFN-γ, provided the co-culture was performed in the medium conditioned by pre-activated ASC and not fresh medium. Intriguingly, pre-treatment with other candidate cytokines tumor necrosis factor (TNF)-α, interleukin (IL)-1β, IL-17, tissue growth factor-β, or stromal cell-derived factor-1α had no reported effect (24).

Little has been reported to date on the use of cytokine-licensed MSC and their immunosuppressive effects on T cell function. Chinnadurai et al., in a recent report, found that only IFN-γ licensed human (h)MSC were able to inhibit the secretion of the key Th1-related cytokines IFN-γ, TNF-α, and IL-2 by allogeneic T cells. In the same experiments, un-licensed MSC had no immunomodulatory effect (23). Moreover, they identified the MSC-expressed, IFN-γ-licensed inhibitory molecules B7H1 and B7DC/programmed death receptor 1 (PD1) pathways as essential effectors in blocking T cell function. The authors also found that, *in vivo*, licensing-induced efficacy was dependent on whether the MSC were used fresh or from frozen (23). While indoleamine 2,3-dioxygenase (IDO) activity was increased in these licensed MSC, its function was largely dispensable with regard to MSC-driven inhibition of T cell effector function (25).

While the inflammatory cytokines IFN-γ, TNF-α, and IL-1β are by the far the most commonly used molecules to license MSC, Sivanathan et al. employed a novel approach to licensing hMSC by pre-activating the cells with IL-17 (MSC-17). Their rationale for this approach was that, while IFN-γ licensed MSC are more immunosuppressive than their un-licensed counterparts they also upregulate MHC I thereby becoming potentially more immunogeneic *in vivo*. Unlike IFN-γ licensed MSC, MSC-17 do not upregulate MHC I (or MHC II), nor the T cell co-stimulatory marker CD40. Furthermore, MSC-17 could inhibit the production of Th1-related cytokines IFN-γ, TNF-α, and IL-2 by allogeneic T cells, in addition to inhibiting surface CD25 expression. In addition, and potentially crucial with regard to therapeutic efficacy, MSC-17 but not IFN-γ licensed MSC consistently induced CD4^+^ CD25^high^ CD127^low^ FoxP3^+^ regulatory T cells (iTregs) from phytohemagglutinin-activated CD4^+^ CD25^−^ T cells (26). As mentioned previously, the majority of studies use licensing as a way to further enhance MSC immunosuppressive ability. However, Roemeling van-Rhijn and colleagues (27) focused on the effect(s) of licensing on the immunogenicity of allogeneic MSC by evaluating whether or not repeated exposure to allogeneic MSC [derived from bone marrow (BM-MSC)] and/or adipose tissue (ASC) induced human leukocyte antigen (HLA) class I specific lysis by CD8^+^ T cells in a human setting. They also tested what effects exposure to IFN-γ had on the MSC and their ability to induce CD8^+^ cytotoxic T cell reactivity. They found that MSC-educated CD8^+^ T cells were able to lyse BM-MSC in an HLA-specific manner. Interestingly, in addition to an observed increase in HLA class I expression, percentage lysis of MSC was doubled when the cells were pre-activated with IFN-γ. Furthermore, co-culture of PBMC with IFN-γ-stimulated BM-MSC further increased percentage lysis. A similar trend was observed when using ASC but percentage lysis was significantly lower compared to BM-MSC (16, 27).

## Immunogenicity and Immunomodulatory Potential of Allogeneic Differentiated MSC

The cell surface expression of MHC proteins and co-stimulatory molecules can have a major impact on allo-recognition of transplanted cells or organs in an immunocompetent host (5). As mentioned previously, MSC are generally thought to express low or no MHC and co-stimulatory proteins contributing to their perceived “weak” immunogenic profile (5). However, as with exposure to a pro-inflammatory environment, MSC differentiation has been previously shown to result in upregulation of cell surface immunogenic molecules (5, 28, 29). In recent years, several studies have examined upregulation of potentially immunogenic cell surface proteins in an allogeneic and xenogeneic cell therapy context. With regard to the effect of MSC differentiation on the cell surface profile of the cells, there are conflicting results presented in different studies. For example, Li et al. describe an upregulation of HLA-DR on MSC after hepatocyte differentiation (30), while several other studies report no change in cell surface proteins after chondrogenic (31), myogenic (32), and insulin-producing cell (33) differentiation.

More important than cell surface expression levels is functional immunogenicity, which is the response of immune cells to the differentiated cells. Differentiated MSC have been shown to elicit allogeneic lymphocyte proliferation after osteogenic (34), chondrogenic (29, 35), and hepatocytic (30) differentiation, and lymphocyte IFN-γ production *in vitro* after hepatocytic differentiation (30). Contrary to these studies, it has also been recently shown that differentiated MSC do not induce allogeneic T cell proliferation *in vitro* after osteogenic (36) or chondrogenic (37) differentiation.

While not directly related to immunogenicity, the secretion of immunoregulatory or immunosuppressive molecules has also been reported as being affected by MSC differentiation. Prostaglandin E_2_ (PGE_2_), an important mediator of MSC immunosuppressive ability has been shown to be reduced after myogenic differentiation (38). On the other hand, it has also been shown that MSC, during chondrogenic differentiation can maintain the expression and production of immunomodulatory molecules, such as nitric oxide, IL-6, and IDO (39).

While *in vitro* data have aided in our understanding of the potential mechanisms occurring during differentiation and the immune response to the transplanted cells, the *in vivo* immune response to the cells is the most indicative of translational potential. Studies that transplanted allogeneic differentiated MSC into appropriate pre-clinical models have shown varying levels of immune response. For example, hepatocyte differentiated MSC induced significantly more CD3^+^ and CD45^+^ cells after transplantation compared to undifferentiated MSC, despite the fact that the differentiated MSC exerted a beneficial effect on the glycemic control of the treated animals (33). Osteogenically differentiated MSC transplantation resulted in significantly more activated immune cells after implantation in a mouse model (34). In a diabetic model, evidence for *in situ* differentiation was observed, increased numbers of cytotoxic cells in recipients and higher levels of allo-antibody were attributed to this differentiation (40).

Much of the evidence discussed here and elsewhere (5, 28, 29) points to an increase in MSC immunogenicity following induced differentiation. Strategies to reduce this immunogenicity, therefore, would be extremely important for the future development of such therapies. Interestingly, Li et al. showed that when they carried out their hepatocyte differentiation in a 3D scaffold, as opposed to 2D tissue culture plastic, they observed no increase in lymphocyte proliferation, no decrease in production of PGE_2_ and less IFN-γ production by lymphocytes (30). The maintenance of expression of immunomodulatory molecules which Yang et al. observed was also only seen in 3D culture conditions as opposed to 2D (39). Gene therapy approaches also hold promise as an approach to reduce the detrimental immune effects of differentiation. Dhingra et al. (38), following up on excellent work from Huang et al. (41), showed that restoration of secretion of PGE_2_ could overcome the rejection and loss of efficacy of cardiomyocyte differentiated MSC (38). It was also shown that interfering with the expression of MHC II on the cell surface of differentiated MSC could increase their survival time *in vivo*, reduce cytotoxic and allo-antibody responses, and increase therapeutic efficacy (14, 42). Our increased understanding of the immunological changes that occur following differentiation of allo-MSC are already informing alternative strategies to circumvent their increased immunogenicity.

## Immunogenicity of Human MSC in Human Subjects

As mentioned earlier, the generation of humoral and/or cellular immune responses against the allogeneic donor cells could potentially lead to adverse immunological effects, impact the efficacy of subsequent allogeneic cell therapy, or compromise the success of future organ transplantation (5). Although it has been shown that allogeneic MSC induce at least a humoral immune response in pre-clinical models, it is not clear whether this occurs on the human setting. To date, approximately 3,000 patients have received allogeneic mesenchymal stem cell treatment for various diseases and no acute adverse events linked to the allogenicity of MSC have been reported (8, 9).

As yet only a limited number of clinical trials have evaluated the generation of potentially harmful anti-HLA antibodies in patients receiving allo-MSCs, and it remains unclear whether this may lead to adverse effects. Four clinical trials have been published that have documented the presence/absence of anti-HLA antibodies in patients after allo-MSC therapy for various disease indications, including osteoarthritis, Crohn’s disease, and Type II diabetes. Results published by Garcia-Sancho et al. on the influence of HLA-matching on the efficacy of allogeneic MSC therapy in osteoarthritis and degenerative disk disease (43), indicated that only a very limited number of patients receiving MSC had developed anti-donor antibodies and, surprisingly, better donor–recipient HLA-matching did not enhance efficacy. In another clinical trial for complex perianal fistulas in Crohn’s disease, Panés et al. reported that allogeneic adipose-derived mesenchymal stem cells (Cx601) treated patients developed donor-specific antibodies (44). At week 12, 34% of Cx601 treated patients generated anti-HLA Class I antibodies compared to none of the placebo-treated group. The authors suggested, however, that the development of donor-specific antibodies had no clinical relevance in terms of affecting the efficacy of Cx601 or in provoking TEAEs. Two other clinical trials have been published by Skyler et al. (45) and Packham et al. (46) using rexlemestrocel-L cells, defined as allogeneic mesenchymal precursor cells (MPC), for the treatment of Type II Diabetes and Diabetic nephropathy. Cross matching between donor cells and recipient was not performed prior to infusion. Both studies indicate that no subjects receiving rexlemestrocel-L treatment developed antibodies specific to the donor HLA or showed clinically relevant increases in either class I or II PRA. The authors suggest that these findings are consistent with the immunotolerant profile of MPCs, which are negative for HLA Class II and CD80 and CD86 co-immunostimulatory molecules, and exert potential immunomodulatory effects, including inhibition of T cell proliferation (47). The observed lack of acute immunological responses to unmatched allogeneic MPC is particularly important in patients who may eventually require organ transplantation. The authors suggest that the lack of any evidence of sustained sensitization and development of antibodies specific to the donor HLA suggests that repeat administration of this therapy may be a feasible option in this patient population.

Taken collectively, these data might indicate that the formation of allo-antibodies may not be as critical as originally thought, though this would likely be dependent on the phenotype of the cells administered as well as disease specificity. However, the implications of the development of donor-specific allo-antibodies will need to be assessed over longer time periods, alongside the tolerability and efficacy of single and repeated administration of allogeneic MSC before definite conclusions can be drawn in this regard.

## Immunogenicity of Extracellular Vesicles Secreted from Allogeneic MSC

In recent years exciting results from the use of MSC-derived extracellular vesicles (EVs) or exosomes has come to light (48–50). This research has been primarily focused on transfer of molecular cargoes, such as mRNA and miRNA (48, 51), to enhance regenerative or pro-survival responses. Furthermore, a recent report form Bai and colleagues (52) could demonstrate the efficacy of human MSC-derived exosomes delivered periocularly in a rat model of experimental autoimmune uveitis (52). However, evidence from dendritic cell research indicates that potentially immunogenic proteins such as MHC molecules can also be transferred *via* EVs (53). This raises the possibility that allogeneic MSC-derived EVs may also possess immunogenic molecules which could lead to potentially detrimental immune responses such as those elicited by allo-MSCs themselves. Although there seems to be evidence that EVs may contain MHC molecules it is not clear at this stage if (i) MSC-derived exosomes contain MHC molecules, (ii) they can be transferred to other cells, and, most notably, (iii) if they may induce allo-immune responses. Another future potential area of interest may be to examine the expression of tissue factor (TF). While TF has been shown to be present in EVs (54), expression has not yet been reported in MSC-derived EVs. Future studies designed to investigate the influence of TF in EVs on anti-donor immune responses or procoagulant activity may yield important insights.

## Perspective

**Figure 1:**
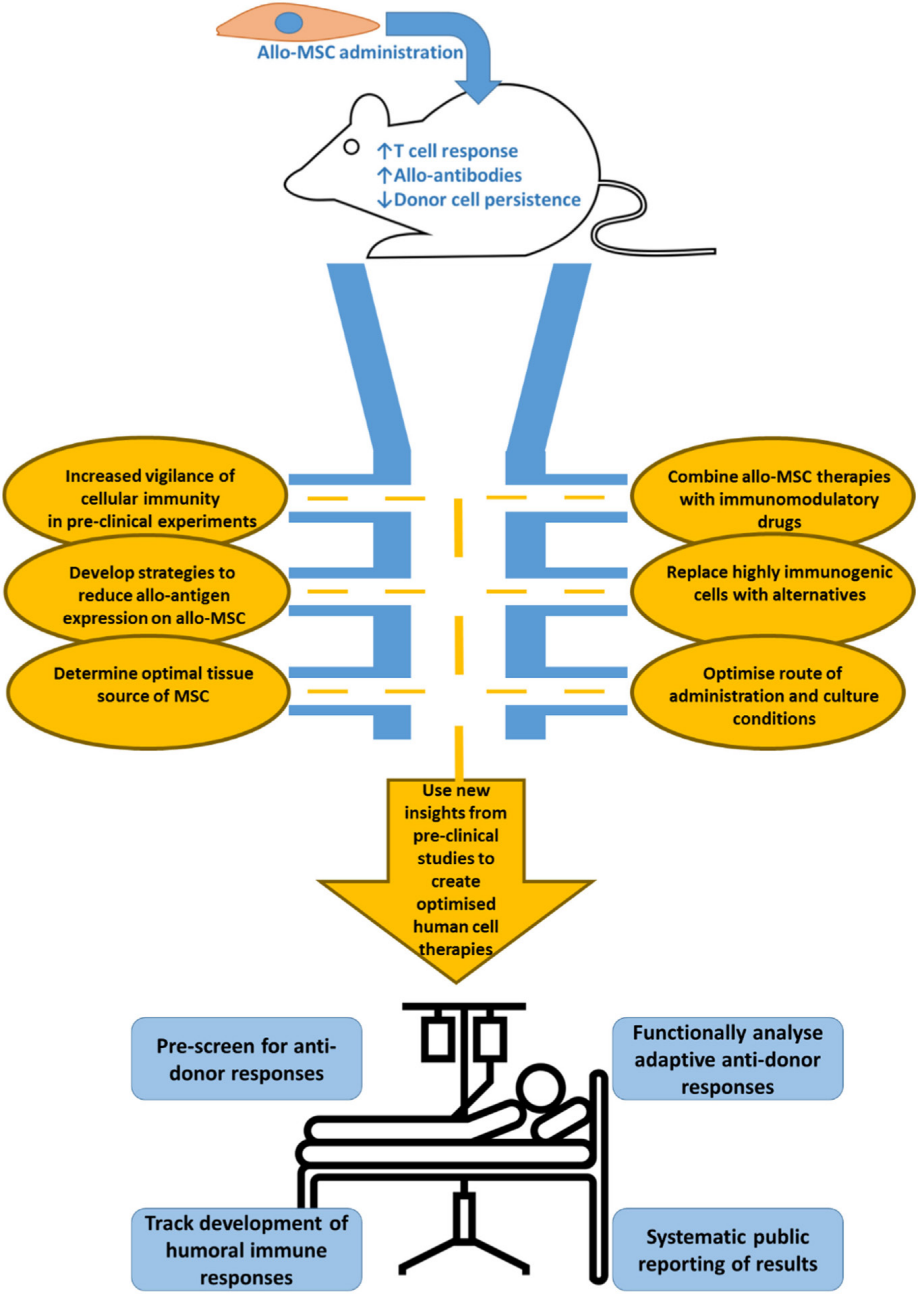
Immunological considerations for the development of next generation allo-MSC therapy. Allogeneic administration of MSC in a variety of pre-clinical models has been shown to induce allo-immune responses. These potentially harmful responses include generation of memory T cells, production of functional allo-antibodies and allo-specific clearance of administered cells. More evidence on the specific effects of allo-MSC administration on a model specific basis is required however and the field would benefit from systematic reporting of relevant allo-immune reactions elicited by MSC in pre-clinical models. The development of the next generation of MSC therapies will require focused and multi-disciplinary pre-clinical experiments to (1) fully characterize and report cell mediated immunity, (2) if necessary, to lower these immune responses in the recipients by working on synergy of allo-MSC with immunomodulatory drugs, (3) by developing strategies to reduce allo-antigen expression on administered cells by using genetic manipulation or small molecules, and (4) if appropriate, replacing highly immunogenic MSC cell therapies with alternatives. In addition to developing bespoke indication specific strategies like those mentioned above, the field as a whole should look to optimize the source, culture conditions and routes of administration of MSC in order to minimize immunogenic reactions to the cells while maintaining efficacy. In the coming years, more detailed pre-clinical data will allow lessons to be learned to create optimized allo-MSC therapies for clinical use. However, we should continue to carefully monitor the allo-immune response once allo-MSC are administered to the patient. This surveillance should include pre-screening patients for pre-existing anti-donor immune responses, monitoring anti-donor cellular and humoral immune responses and or equal importance systematic reporting of results in easily available formats.

While in recent years strong evidence has emerged showing that allogeneic MSC can and do invoke measurable immune responses, there is still ambiguity and uncertainty in the field. The next generation of allo-MSC therapies should be developed through rigorous characterization and fine tuning of MSC immunogenicity, survival, and persistence after transplantation, efficacy, and disease-specific mechanisms of action (Figure 1).

Consideration of the points below is likely to lead to considerable advances in our understanding of the anti-donor immune responses induced by allogeneic MSC therapy and potential new strategies to minimize any potential adverse responses while maximizing patient benefit,

1. Establishment and monitoring of safety/efficacy profiles for each specific treatment regimen in different treatment indications and disease stages to evaluate, for example, the risk of allo-immunization relative to the beneficial effects of the treatment.
2. Comprehensive analysis of the full range of characteristics affecting immunogenicity such as cell surface expression of important molecules on the MSC, *in vitro* responses of dendritic, T, and B cells and *in vivo* humoral, cytotoxic, and memory responses.
3. The use of immunosuppressive drugs or other molecules in combination with allogeneic MSC to determine if the immune response to the cells can be reduced.
4. Use of gene therapy or small molecule approaches to interfere with the presentation of allo-antigen on the cell surface.
5. Replacement of highly immunogenic cells such as some differentiated MSC (29). Alternative cell types could be investigated to replace damaged tissue (55, 56), or in-depth studies into the mechanism of action of, e.g., licensed MSC may yield candidate molecules which could replace cell therapy altogether.
6. Investigation of alternative routes of administration (e.g., “inert” locations such as the articular cavity or intervertebral disk (43) which may lower the “visibility” of the cells to the host immune system.
7. A detailed understanding of how allo-MSC cell death affects their immunogenic or tolerogenic properties and how (or if) allo-MSC cell death regulates ensuing anti-donor immune responses.

It is clear that MSC therapy, and particularly allogeneic MSC therapy, holds great promise for the treatment of a multitude of diseases. These cells have been shown to be clinically safe despite abundant pre-clinical evidence that they invoke an anti-donor immune response. The systematic collection and dissemination of immunological data should still be undertaken to provide further insight into mechanism of action and so that researchers, clinicians, and regulatory bodies worldwide can remain vigilant to any future adverse effects.

## Author Contributions

PL, OT, and AR wrote the manuscript and finalized the draft. MG and TR wrote specific parts of the manuscript and revised the entire draft.

## Conflict of Interest Statement

The authors declare that the research was conducted in the absence of any commercial or financial relationships that could be construed as a potential conflict of interest. The reviewer DN and handling editor declared their shared affiliation.

## References

[1] Caplan AI. Mesenchymal stem cells: time to change the name! Stem Cells Transl Med (2017) 6(6):1445–51. 10.1002/sctm.17-0051 28452204 PMC5689741

[2] Di Nicola M Carlo-Stella C Magni M Milanesi M Longoni PD Matteucci P Human bone marrow stromal cells suppress T-lymphocyte proliferation induced by cellular or nonspecific mitogenic stimuli. Blood (2002) 99(10):3838–43. 10.1182/blood.V99.10.3838 11986244

[3] Barry FP Murphy JM English K Mahon BP. Immunogenicity of adult mesenchymal stem cells: lessons from the fetal allograft. Stem Cells Dev (2005) 14(3):252–65. 10.1089/scd.2005.14.252 15969620

[4] Eliopoulos N Stagg J Lejeune L Pommey S Galipeau J. Allogeneic marrow stromal cells are immune rejected by MHC class I- and class II-mismatched recipient mice. Blood (2005) 106(13):4057–65. 10.1182/blood-2005-03-1004 16118325

[5] Griffin MD Ryan AE Alagesan S Lohan P Treacy O Ritter T. Anti-donor immune responses elicited by allogeneic mesenchymal stem cells: what have we learned so far? Immunol Cell Biol (2013) 91(1):40–51. 10.1038/icb.2012.67 23207278

[6] Nauta AJ Westerhuis G Kruisselbrink AB Lurvink EG Willemze R Fibbe WE. Donor-derived mesenchymal stem cells are immunogenic in an allogeneic host and stimulate donor graft rejection in a nonmyeloablative setting. Blood (2006) 108(6):2114–20. 10.1182/blood-2005-11-011650 16690970 PMC1895546

[7] Schu S Nosov M O’Flynn L Shaw G Treacy O Barry F Immunogenicity of allogeneic mesenchymal stem cells. J Cell Mol Med (2012) 16(9):2094–103. 10.1111/j.1582-4934.2011.01509.x 22151542 PMC3822979

[8] Le Blanc K Frassoni F Ball L Locatelli F Roelofs H Lewis I Mesenchymal stem cells for treatment of steroid-resistant, severe, acute graft-versus-host disease: a phase II study. Lancet (2008) 371(9624):1579–86. 10.1016/S0140-6736(08)60690-X 18468541

[9] Gregoire C Lechanteur C Briquet A Baudoux E Baron F Louis E Review article: mesenchymal stromal cell therapy for inflammatory bowel diseases. Aliment Pharmacol Ther (2017) 45(2):205–21. 10.1111/apt.13864 27878827

[10] Kaipe H Carlson LM Erkers T Nava S Mollden P Gustafsson B Immunogenicity of decidual stromal cells in an epidermolysis bullosa patient and in allogeneic hematopoietic stem cell transplantation patients. Stem Cells Dev (2015) 24(12):1471–82. 10.1089/scd.2014.0568 25658253 PMC4485366

[11] Baygan A Aronsson-Kurttila W Moretti G Tibert B Dahllof G Klingspor L Safety and side effects of using placenta-derived decidual stromal cells for graft-versus-host disease and hemorrhagic cystitis. Front Immunol (2017) 8:795. 10.3389/fimmu.2017.00795 28744284 PMC5504152

[12] Melmed GY Pandak WM Casey K Abraham B Valentine J Schwartz D Human placenta-derived cells (PDA-001) for the treatment of moderate-to-severe Crohn’s disease: a phase 1b/2a study. Inflamm Bowel Dis (2015) 21(8):1809–16. 10.1097/MIB.0000000000000441 25985246

[13] Moll G Ignatowicz L Catar R Luecht C Sadeghi B Hamad O Different procoagulant activity of therapeutic mesenchymal stromal cells derived from bone marrow and placental decidua. Stem Cells Dev (2015) 24(19):2269–79. 10.1089/scd.2015.0120 26192403

[14] Ankrum JA Ong JF Karp JM. Mesenchymal stem cells: immune evasive, not immune privileged. Nat Biotechnol (2014) 32(3):252–60. 10.1038/nbt.2816 24561556 PMC4320647

[15] Schnabel LV Pezzanite LM Antczak DF Felippe MJ Fortier LA. Equine bone marrow-derived mesenchymal stromal cells are heterogeneous in MHC class II expression and capable of inciting an immune response in vitro. Stem Cell Res Ther (2014) 5(1):13. 10.1186/scrt402 24461709 PMC4055004

[16] Gotherstrom C Ringden O Tammik C Zetterberg E Westgren M Le Blanc K Immunologic properties of human fetal mesenchymal stem cells. Am J Obstet Gynecol (2004) 190(1):239–45. 10.1016/j.ajog.2003.07.022 14749666

[17] Joswig AJ Mitchell A Cummings KJ Levine GJ Gregory CA Smith R III Repeated intra-articular injection of allogeneic mesenchymal stem cells causes an adverse response compared to autologous cells in the equine model. Stem Cell Res Ther (2017) 8(1):42. 10.1186/s13287-017-0503-8 28241885 PMC5329965

[18] Berglund AK Schnabel LV. Allogeneic major histocompatibility complex-mismatched equine bone marrow-derived mesenchymal stem cells are targeted for death by cytotoxic anti-major histocompatibility complex antibodies. Equine Vet J (2017) 49(4):539–44. 10.1111/evj.12647 27862236 PMC5425313

[19] Ardanaz N Vazquez FJ Romero A Remacha AR Barrachina L Sanz A Inflammatory response to the administration of mesenchymal stem cells in an equine experimental model: effect of autologous, and single and repeat doses of pooled allogeneic cells in healthy joints. BMC Vet Res (2016) 12:65. 10.1186/s12917-016-0692-x 27029614 PMC4815220

[20] Isakova IA Lanclos C Bruhn J Kuroda MJ Baker KC Krishnappa V Allo-reactivity of mesenchymal stem cells in rhesus macaques is dose and haplotype dependent and limits durable cell engraftment in vivo. PLoS One (2014) 9(1):e87238. 10.1371/journal.pone.0087238 24489878 PMC3906169

[21] Green DR Ferguson T Zitvogel L Kroemer G. Immunogenic and tolerogenic cell death. Nat Rev Immunol (2009) 9(5):353–63. 10.1038/nri2545 19365408 PMC2818721

[22] Moll G Geissler S Catar R Ignatowicz L Hoogduijn MJ Strunk D Cryopreserved or fresh mesenchymal stromal cells: only a matter of taste or key to unleash the full clinical potential of MSC therapy? Adv Exp Med Biol (2016) 951:77–98. 10.1007/978-3-319-45457-3_7 27837556

[23] Chinnadurai R Copland IB Garcia MA Petersen CT Lewis CN Waller EK Cryopreserved mesenchymal stromal cells are susceptible to T-cell mediated apoptosis which is partly rescued by IFNgamma licensing. Stem Cells (2016) 34(9):2429–42. 10.1002/stem.2415 27299362 PMC5016228

[24] Mancheno-Corvo P Menta R del Rio B Franquesa M Ramirez C Hoogduijn MJ T lymphocyte prestimulation impairs in a time-dependent manner the capacity of adipose mesenchymal stem cells to inhibit proliferation: role of interferon gamma, poly I:C, and tryptophan metabolism in restoring adipose mesenchymal stem cell inhibitory effect. Stem Cells Dev (2015) 24(18):2158–70. 10.1089/scd.2014.0508 26058889

[25] Chinnadurai R Copland IB Patel SR Galipeau J IDO-independent suppression of T cell effector function by IFN-gamma-licensed human mesenchymal stromal cells. J Immunol (2014) 192(4):1491–501. 10.4049/jimmunol.1301828 24403533

[26] Sivanathan KN Rojas-Canales DM Hope CM Krishnan R Carroll RP Gronthos S Interleukin-17A-induced human mesenchymal stem cells are superior modulators of immunological function. Stem Cells (2015) 33(9):2850–63. 10.1002/stem.2075 26037953

[27] Roemeling-van Rhijn M Reinders ME Franquesa M Engela AU Korevaar SS Roelofs H Human allogeneic bone marrow and adipose tissue derived mesenchymal stromal cells induce CD8+ cytotoxic T cell reactivity. J Stem Cell Res Ther (2013) 3(Suppl 6):004. 10.4172/2157-7633.S6-004 24729944 PMC3982127

[28] Lohan P Coleman CM Murphy JM Griffin MD Ritter T Ryan AE. Changes in immunological profile of allogeneic mesenchymal stem cells after differentiation: should we be concerned? Stem Cell Res Ther (2014) 5(4):99. 10.1186/scrt488 25158057 PMC4282147

[29] Ryan AE Lohan P O’Flynn L Treacy O Chen X Coleman C Chondrogenic differentiation increases antidonor immune response to allogeneic mesenchymal stem cell transplantation. Mol Ther (2014) 22(3):655–67. 10.1038/mt.2013.261 24184966 PMC3944342

[30] Li Y Wu Q Wang Y Li L Chen F Shi Y Immunogenicity of hepatic differentiated human umbilical cord mesenchymal stem cells promoted by porcine decellularized liver scaffolds. Xenotransplantation (2017) 24(1). 10.1111/xen.12287 28102609

[31] Lee HJ Kang KS Kang SY Kim HS Park SJ Lee SY Immunologic properties of differentiated and undifferentiated mesenchymal stem cells derived from umbilical cord blood. J Vet Sci (2016) 17(3):289–97. 10.4142/jvs.2016.17.3.289 26726028 PMC5037295

[32] Joo S Lim HJ Jackson JD Atala A Yoo JJ. Myogenic-induced mesenchymal stem cells are capable of modulating the immune response by regulatory T cells. J Tissue Eng (2014) 5:2041731414524758. 10.1177/2041731414524758 24555015 PMC3927963

[33] Yang XF Chen T Ren LW Yang L Qi H Li FR. Immunogenicity of insulin-producing cells derived from human umbilical cord mesenchymal stem cells. Exp Ther Med (2017) 13(4):1456–64. 10.3892/etm.2017.4096 28413492 PMC5377284

[34] Fu X Yang H Zhang H Wang G Liu K Gu Q Improved osteogenesis and upregulated immunogenicity in human placenta-derived mesenchymal stem cells primed with osteogenic induction medium. Stem Cell Res Ther (2016) 7(1):138. 10.1186/s13287-016-0400-6 27649692 PMC5028975

[35] Mukonoweshuro B Brown CJ Fisher J Ingham E Immunogenicity of undifferentiated and differentiated allogeneic mouse mesenchymal stem cells. J Tissue Eng (2014) 5:2041731414534255 10.1177/2041731414534255 24812582 PMC4014080

[36] Niu J Ding G Zhang L. Effects of simvastatin on the osteogenic differentiation and immunomodulation of bone marrow mesenchymal stem cells. Mol Med Rep (2015) 12(6):8237–40. 10.3892/mmr.2015.4476 26499955

[37] Kiernan CH Hoogduijn MJ Franquesa M Wolvius EB Brama PA Farrell E. Allogeneic chondrogenically differentiated human mesenchymal stromal cells do not induce immunogenic responses from T lymphocytes in vitro. Cytotherapy (2016) 18(8):957–69. 10.1016/j.jcyt.2016.05.002 27288309

[38] Dhingra S Li P Huang XP Guo J Wu J Mihic A Preserving prostaglandin E2 level prevents rejection of implanted allogeneic mesenchymal stem cells and restores postinfarction ventricular function. Circulation (2013) 128(11 Suppl 1):S69–78. 10.1161/CIRCULATIONAHA.112.000324 24030423

[39] Yang J Chen X Yuan T Yang X Fan Y Zhang X. Regulation of the secretion of immunoregulatory factors of mesenchymal stem cells (MSCs) by collagen-based scaffolds during chondrogenesis. Mater Sci Eng C Mater Biol Appl (2017) 70(Pt 2):983–91. 10.1016/j.msec.2016.04.096 27772730

[40] Gu LH Zhang TT Li Y Yan HJ Qi H Li FR. Immunogenicity of allogeneic mesenchymal stem cells transplanted via different routes in diabetic rats. Cell Mol Immunol (2015) 12(4):444–55. 10.1038/cmi.2014.70 25242276 PMC4496541

[41] Huang XP Sun Z Miyagi Y McDonald Kinkaid H Zhang L Weisel RD Differentiation of allogeneic mesenchymal stem cells induces immunogenicity and limits their long-term benefits for myocardial repair. Circulation (2010) 122(23):2419–29. 10.1161/CIRCULATIONAHA.110.955971 21098445

[42] Huang XP Ludke A Dhingra S Guo J Sun Z Zhang L Class II transactivator knockdown limits major histocompatibility complex II expression, diminishes immune rejection, and improves survival of allogeneic bone marrow stem cells in the infarcted heart. FASEB J (2016) 30(9):3069–82. 10.1096/fj.201600331R 27221978

[43] Garcia-Sancho J Sanchez A Vega A Noriega DC Nocito M. Influence of HLA matching on the efficacy of allogeneic mesenchymal stromal cell therapies for osteoarthritis and degenerative disc disease. Transplant Direct (2017) 3(9):e205. 10.1097/TXD.0000000000000724 28894792 PMC5585421

[44] Panes J Garcia-Olmo D Van Assche G Colombel JF Reinisch W Baumgart DC Expanded allogeneic adipose-derived mesenchymal stem cells (Cx601) for complex perianal fistulas in Crohn’s disease: a phase 3 randomised, double-blind controlled trial. Lancet (2016) 388(10051):1281–90. 10.1016/S0140-6736(16)31203-X 27477896

[45] Skyler JS Fonseca VA Segal KR Rosenstock J Investigators M-D. Allogeneic mesenchymal precursor cells in type 2 diabetes: a randomized, placebo-controlled, dose-escalation safety and tolerability pilot study. Diabetes Care (2015) 38(9):1742–9. 10.2337/dc14-2830 26153271 PMC4542273

[46] Packham DK Fraser IR Kerr PG Segal KR. Allogeneic mesenchymal precursor cells (MPC) in diabetic nephropathy: a randomized, placebo-controlled, dose escalation study. EBioMedicine (2016) 12:263–9. 10.1016/j.ebiom.2016.09.011 27743903 PMC5078602

[47] Togel F Westenfelder C The role of multipotent marrow stromal cells (MSCs) in tissue regeneration. Organogenesis (2011) 7(2):96–100. 10.4161/org.7.2.15781 21521944 PMC3142445

[48] Figueroa J Phillips LM Shahar T Hossain A Gumin J Kim H Exosomes from glioma-associated mesenchymal stem cells increase the tumorigenicity of glioma stem-like cells via transfer of miR-1587. Cancer Res (2017) 77(21):5808–19. 10.1158/0008-5472.CAN-16-2524 28855213 PMC5668150

[49] Cheng X Zhang G Zhang L Hu Y Zhang K Sun X Mesenchymal stem cells deliver exogenous miR-21 via exosomes to inhibit nucleus pulposus cell apoptosis and reduce intervertebral disc degeneration. J Cell Mol Med (2017). 10.1111/jcmm.13316 PMC5742691 28805297

[50] Shimoda A Tahara Y Sawada SI Sasaki Y Akiyoshi K. Glycan profiling analysis using evanescent-field fluorescence-assisted lectin array: importance of sugar recognition for cellular uptake of exosomes from mesenchymal stem cells. Biochem Biophys Res Commun (2017) 491(3):701–7. 10.1016/j.bbrc.2017.07.126 28751214

[51] Xin H Li Y Chopp M. Exosomes/miRNAs as mediating cell-based therapy of stroke. Front Cell Neurosci (2014) 8:377. 10.3389/fncel.2014.00377 25426026 PMC4226157

[52] Bai L Shao H Wang H Zhang Z Su C Dong L Effects of mesenchymal stem cell-derived exosomes on experimental autoimmune uveitis. Sci Rep (2017) 7(1):4323. 10.1038/s41598-017-04559-y 28659587 PMC5489510

[53] Liu Q Rojas-Canales DM Divito SJ Shufesky WJ Stolz DB Erdos G Donor dendritic cell-derived exosomes promote allograft-targeting immune response. J Clin Invest (2016) 126(8):2805–20. 10.1172/JCI84577 27348586 PMC4966303

[54] Park JA Sharif AS Tschumperlin DJ Lau L Limbrey R Howarth P Tissue factor-bearing exosome secretion from human mechanically stimulated bronchial epithelial cells in vitro and in vivo. J Allergy Clin Immunol (2012) 130(6):1375–83. 10.1016/j.jaci.2012.05.031 22828416 PMC3511625

[55] Lohan P Treacy O Lynch K Barry F Murphy M Griffin MD Culture expanded primary chondrocytes have potent immunomodulatory properties and do not induce an allogeneic immune response. Osteoarthritis Cartilage (2016) 24(3):521–33. 10.1016/j.joca.2015.10.005 26493330

[56] Huey DJ Sanchez-Adams J Willard VP Athanasiou KA. Immunogenicity of bovine and leporine articular chondrocytes and meniscus cells. Tissue Eng Part A (2012) 18(5–6):568–75. 10.1089/ten.TEA.2011.0226 21942992 PMC3286814

